# Innovative hybrid approach: digital and analog fabrication of orbital prosthesis for post-COVID-19 mucormycosis defects using photogrammetry technique

**DOI:** 10.1038/s41598-024-77836-2

**Published:** 2024-11-02

**Authors:** Akansha Vilas Bansod, Sweta Kale Pisulkar, Arushi Beri, Ritul Jain, Shruti Deshmukh, Utkarsh Umre

**Affiliations:** 1 Department of Prosthodontics and Crown and Bridge, Sharad Pawar Dental College and Hospital, Datta Meghe Institute of Higher Education and Research, Sawangi (M) Wardha, 442001 Maharashtra India; 2 Department of Conservative Dentistry and Endodontics, Sharad Pawar Dental College and Hospital, Datta Meghe Institute of Higher Education and Research, Sawangi (M) Wardha, 442001 Maharashtra India

**Keywords:** Orbital prosthesis, Digital workflow, COVID-19, Mucormycosis, Photogrammetry, Gastroenterology, Health care, Medical research

## Abstract

In addition to anatomical loss, removal of the full or portion of the orbit results in a facial deformity and psychological distress for the patient. This article details a practical case of prosthetic rehabilitation using digital workflow for an orbital deformity caused by post-COVID-19 Mucor mycosis. The main goal of this case study was to create a maxillofacial orbital prosthesis that is well-retained, simple to use, and accurate in terms of appearance. The study addresses the problems involved in fabricating the orbital prosthesis, particularly the unique dimensions and form of the defect, replicating the natural skin tone, and accomplishing retention by the most prudent and patient-friendly approach. Through this article, a digitised algorithm, using photogrammetry technique for facial scan, is suggested for fabricating the prosthesis.

## Introduction

Exenteration of orbital contents has a negative psychological and cosmetic influence on the patient^1^. It is used to treat chronically developing ocular diseases that are unresponsive to prior treatment or eye tumours that could be fatal. Due to the recent increase in COVID-19 instances, patients were seen to be suffering from severe opportunistic fungal infections, including rhino-orbito-cerebral mucormycosis. India has had higher mortality as well as morbidity as a result of the fungus mucormycosis during the second wave^2^.

Orbital defects can be treated with surgical reconstruction procedure and maxillofacial prostheses. Surgical procedures cannot completely restore the anatomical structures of the orbital region or the ocular globe once they have been resected. Surgical interventions are limited to closing defects rather than reconstructing the original anatomy. In contrast, prosthetic rehabilitation offers the advantage of creating prostheses that closely resemble natural features without the need for extensive surgical procedures. This approach facilitates regular clinical examinations of the affected area^3^.

Both analogue and digital techniques have been discussed in literature over years^4^. Conventional has its own drawbacks, such as poor retention because the defect is difficult to access, laborious, time-consuming, and requiring skilled professionals, whereas digital is more affluent but also more accurate in terms of surface detail recordings and technique sensitivity^5^. Methods for digital scanning technique involves using 3D scanning and a virtual digital model is created from the point cloud data. Photogrammetry is a second method for creating 3D digital models from finished images taken from different angles. Both methods are contactless and time-saving compared to traditional data collection methods. 3D scanning is a fast and accurate process, but it has disadvantages such as high installation costs and the need for specialized personnel. Due to these shortcomings, this technique has had very limited application in prosthetic design. With a slight compromise on the accuracy of the 3D model, photogrammetry can overcome the shortcomings of its 3D scanning. Among other things, the availability of free software for modelling, the ability that even a skilled salesperson can handle the process, and the fact that even a cell phone camera Working with standard operating procedures can also achieve good results.

The accuracy of the photogrammetry technique applied to gypsum models, obtained from conventional impressions was thoroughly evaluated by Beri et al., as documented in their study^6^. The photogrammetry technique, based on the structure-from-motion algorithm, converts multiple high-resolution images taken from different angles into a 3D model^7^. Beri at al. conducted calibration study to determine the impact of the number of photos on the quality of the resulting 3D models. Photographs were taken in sets of 90, 60, and 30 for each gypsum model. Autodesk Recap, a web-based software, was utilized for processing; users only needed to upload the images, and the software handled all subsequent procedures, including noise removal and hole closure, before exporting the models in high-quality STL(Stereolithography**)** format^6^.

For reference, 3D models obtained via CT scan were used. The study compared the results based on four parameters: total time (acquisition time plus processing time), accuracy, completeness, and resolution. Among the different sets, photogrammetry using a DSLR camera with 90 photos yielded the highest accuracy for the gypsum models. There is a practical knowledge gap in the study conducted by Beri et al., as it was performed on gypsum models rather than on actual patients^3,6^. For the technique to be practical and feasible for patient prostheses fabrication, further studies on patients are necessary. Translating from pre-clinical research, we have reached to phase 2 of translational research. Many previous studies have focused on the theoretical aspects of photogrammetry, with very few addressing its practical application. Therefore, our study is a valuable contribution to practical research on the final prostheses fabricated using this technique.

In this article, we propose a hybrid process that employs photogrammetry to scan the defect, compares it with the gold standard CT scan model, and then uses 3D printing to fabricate the prosthesis. This approach combines the advantages of both analogue and digital methods. As a result, we have created an orbital prosthesis that is well-retained, user-friendly, and aesthetically pleasing.

## Materials and methods

The approval for the study is taken from institutional ethics committee (Institutional ethical committee of Data Meghe Institute of Higher Education and Research with IEC No. IEC 2022/781 and registered in Clinical Trial registry of India, CTRI Number CTRI/2022/08/044524 on date 01/08/2022. All methods were carried out in accordance with relevant guidelines and regulations and informed consent was obtained from the subject.

Table 1 includes the case information, including the patient’s age, a summary of the case history, the clinical findings, the surgical procedure, and the anticipated prosthetic rehabilitation.

**Table 1:** Brief history of the patient.

Age/gender	65/ male
Brief case history	Chief Complaint: The patient presented with aesthetic concerns regarding facial deformity approximately seven months after undergoing left eye exenteration. His medical history includes a six-month period of oral medication for COVID-19-induced type II diabetes. Additionally, he tested positive for COVID-19, requiring a three-month hospitalisation; however, the patient was not vaccinated against the virus. According to clinical assessment, the left eye’s exenteration socket has healed satisfactorily. Histopathological analysis revealed wide-angle branching in non-septate hyaline hyphae, indicative of mucormycosis. Imaging studies, including CT, MRI of the paranasal sinuses, brain, and orbit, showed cellulitis of the left orbit, left cavernous sinus, and left superior ophthalmic vein thrombosis, which may be associated with mucormycosis in the setting of invasive fungal pansinusitis on the left side
Affected site	Left eye **(**Fig. 1**)**
Orbital defect shape/size	Roughly Oval, 3.5 cm medio-laterally and 3 cm supero-inferiorly. **(**Fig. 2**)**
Surgical intervention	Left orbital exenteration
Maxillofacial prosthetic rehabilitation	Using maxillofacial silicone, a left orbital prosthesis with tissue undercuts and spectacles for retention

**Fig. 1:**
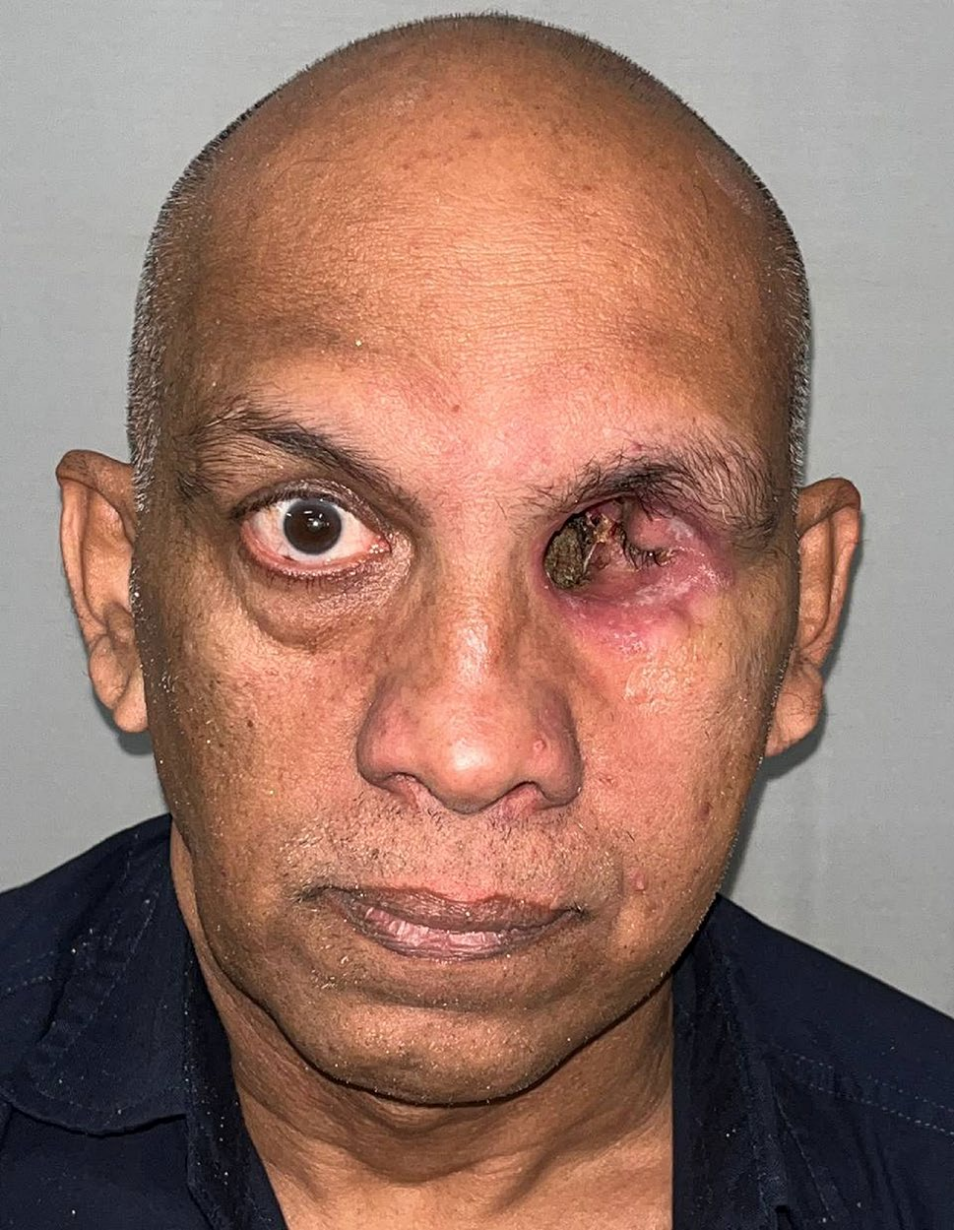
Pre-op photograph of the orbital defect created after surgical removal.

**Fig. 2:**
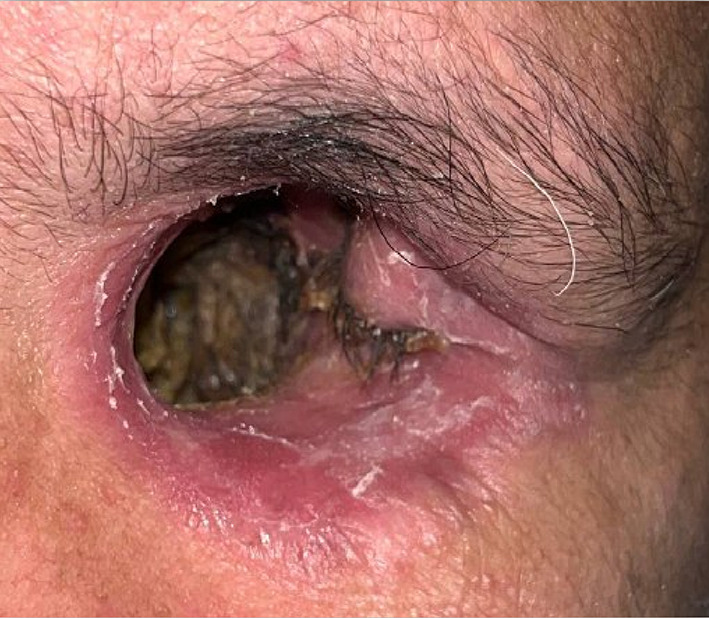
Close-up view of the left orbital defect.

### Data acquisition and processing

#### Defect scan

**Fig. 3:**
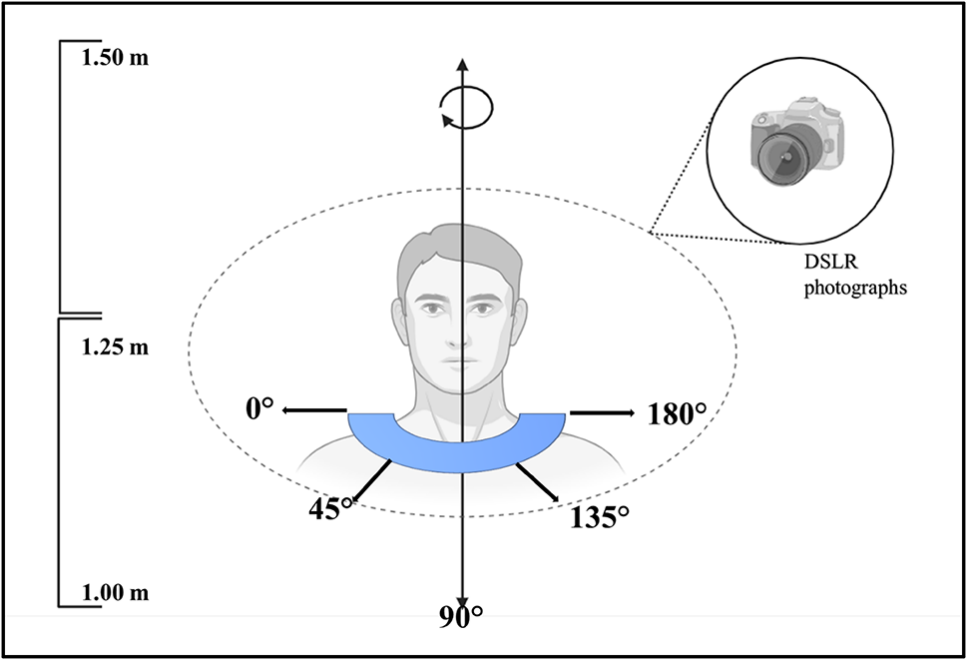
Simulation of angle and sequence of photo capture protocol at three different heights and angles for photogrammetry data acquisition.

The patient was seated on a turntable with a continuous light source to ensure evenly distributed illumination and avoid shadows on the subject. A Nikon D5300 DSLR camera was mounted on a tripod to minimize motion blur during image acquisition. To capture every detail of the defect area on the patient’s face, photographs were taken from three different elevations—lower, mid, and upper—targeting the defect. The camera positions are illustrated in Fig. 3. A total of 90 photographs were taken. Data was processed using Agisoft metashape, an open-source software, to align and merge the images.

**Fig. 4:**
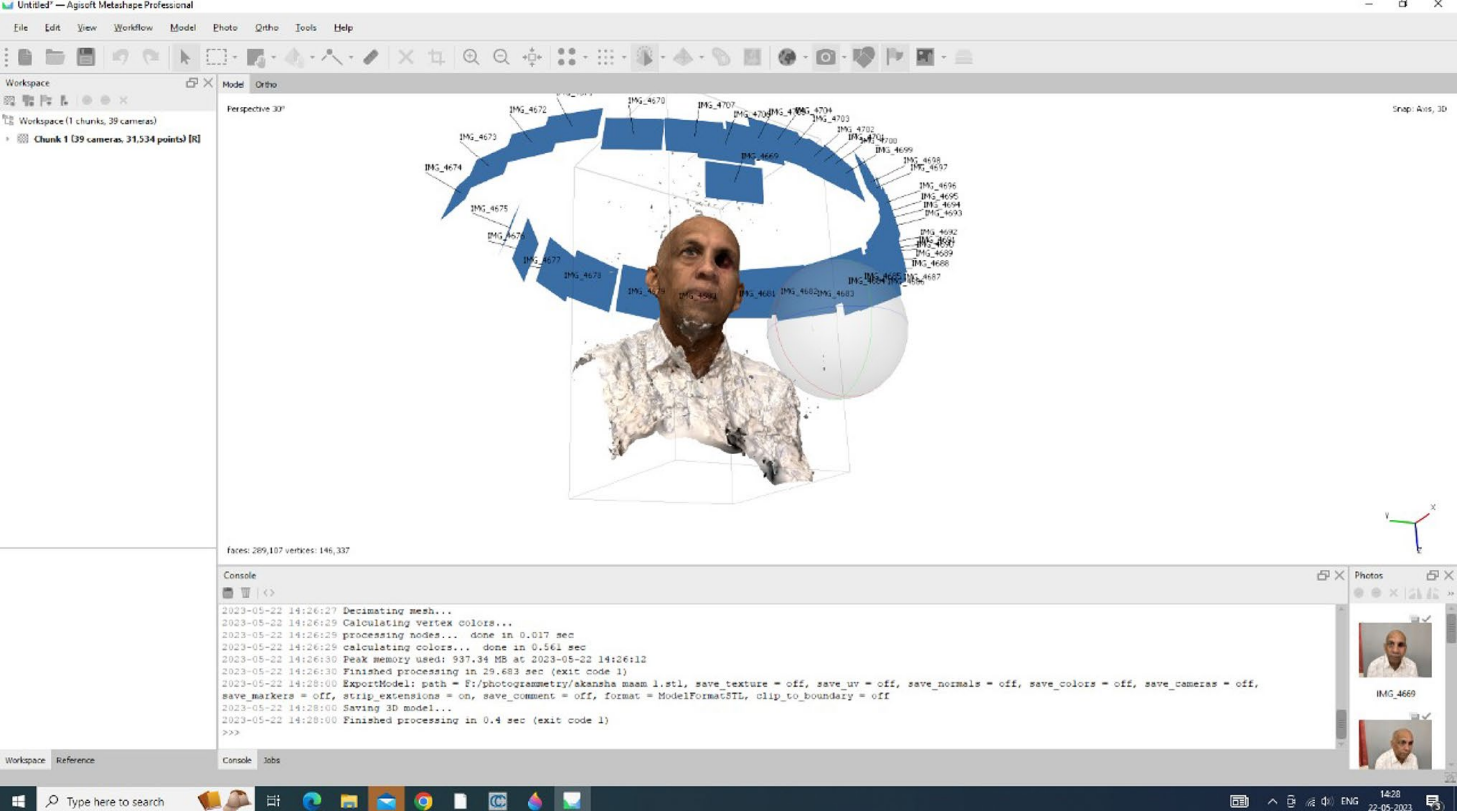
Photogrammetry scan data aligned using agisoft metashape software (Agisoft Metashape 2.1.3 https://agisoft.com/).

Facial model: This data was the converted to STL file which was then utilized to generate a 3D model of the patient’s face. Subsequently, for Rapid Prototyping the model, STL files were employed with model resin, and a model was produced using a 3D printer (Free Shape, manufactured by Akcuretta, Australia). **(**Fig. 4**)**

**Fig. 5:**
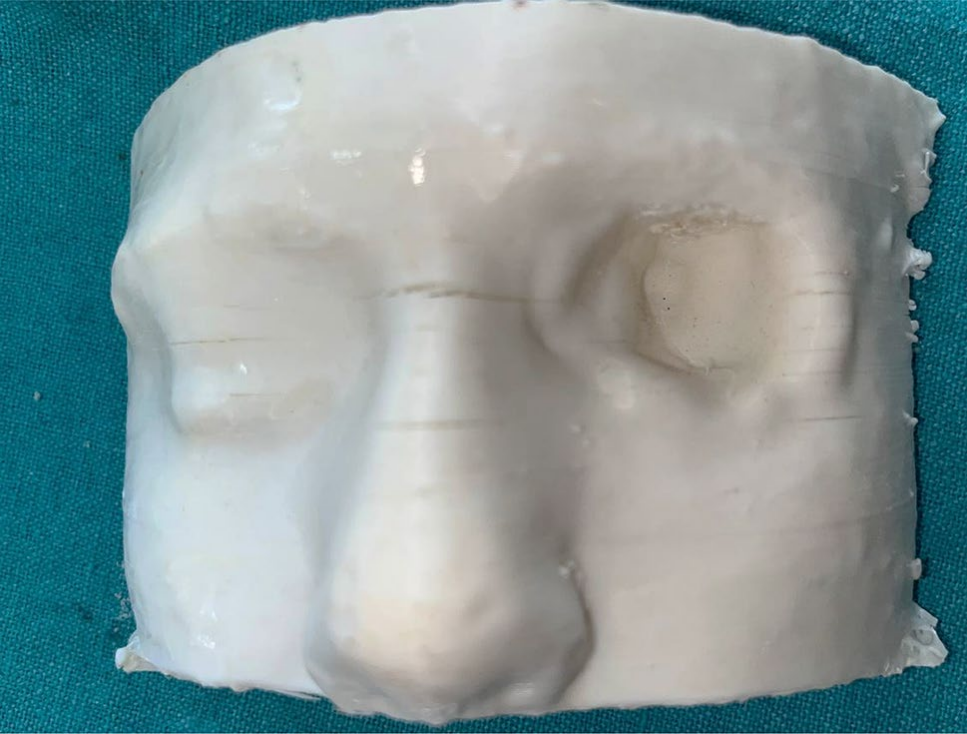
3D-printed working model or a 3D face model.

 Fabrication of trial prosthesis: The 3D-printed working model or a 3D face model (Fig. 5) was employed to create a wax tissue surface for the trial prosthesis fabrication. During this phase, the stability and retention of the prosthesis within the defect were re-evaluated and confirmed.

### Figure 5 3D printed model

**Fig. 6:**
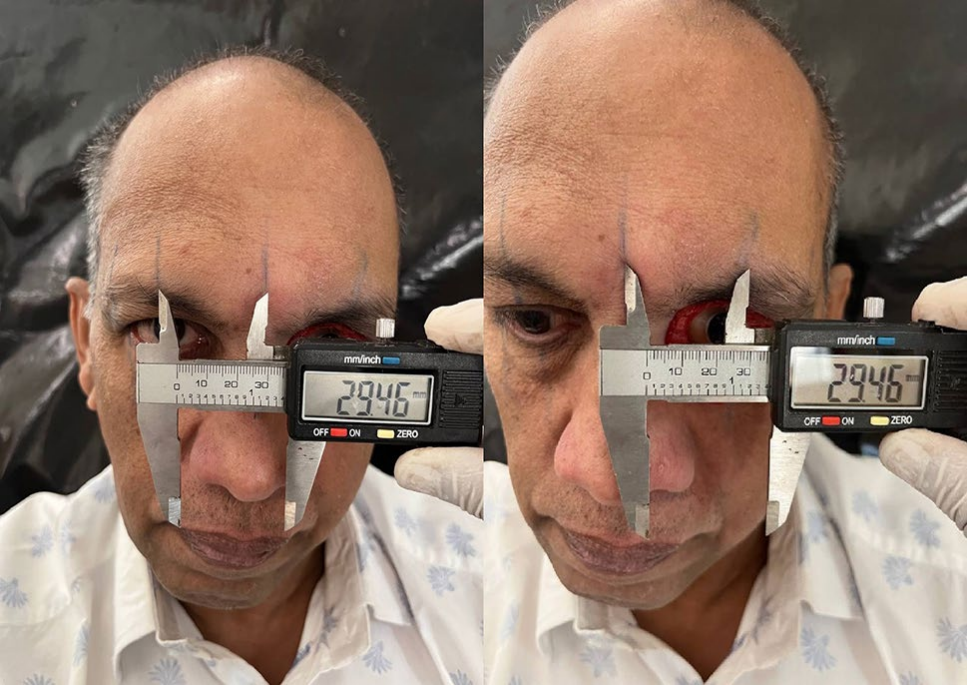
Facial measurements and iris alignment.

**Fig. 7:**
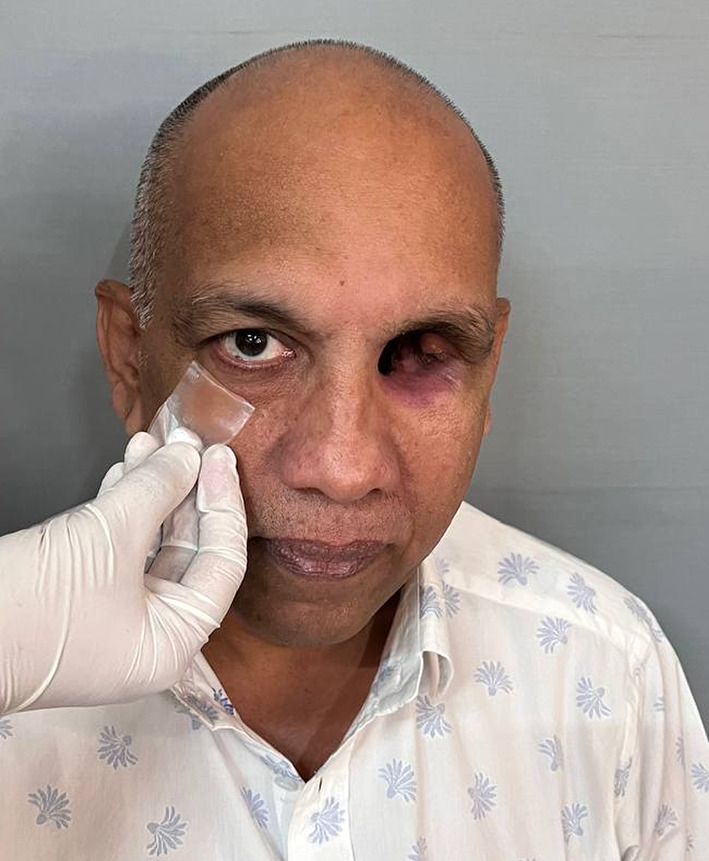
Silicone shade matching.

The wax pattern was meticulously carved and polished based on the facial dimensions obtained. To enhance readers’ understanding of the **iris positioning** process in the manuscript, we have elaborated on several key aspects. First, we emphasize the importance of accurate iris placement, as it is crucial for achieving a natural and aesthetically pleasing appearance in the final wax mold. Proper alignment ensures that the iris corresponds correctly with surrounding anatomical features, contributing to the overall realism of the prosthetic.In our process, we employed vernier calipers to obtain precise facial measurements, allowing for highly accurate readings that replicate the dimensions of the patient’s anatomy in the wax model. This measurement procedure involved several steps. Initially, we identified key anatomical landmarks on the patient’s face, such as the outer and inner canthi, which served as reference points for determining the expected position of the iris. Using the digital vernier calipers, we took measurements from these reference points to establish the exact location for iris placement, as well as the size of the iris itself. All measurements were meticulously recorded to ensure accuracy during the wax pattern fabrication.Once the measurements were obtained, we conducted meticulous iris alignment. The wax pattern was first shaped and prepared to ensure a suitable surface for attachment. The iris was then positioned in accordance with the recorded measurements, carefully aligning it with the established reference points. This step required attention to detail to ensure that the iris was not only accurately placed but also harmonized with the overall anatomy of the model. After placement, we verified the positioning by comparing it to the original measurements and surrounding anatomical features, ensuring accuracy before finalizing the wax pattern. (Fig. 6) Silicone shade matching was done to match the natural skin tone, under natural day-light. (Fig. 7)

**Fig. 8:**
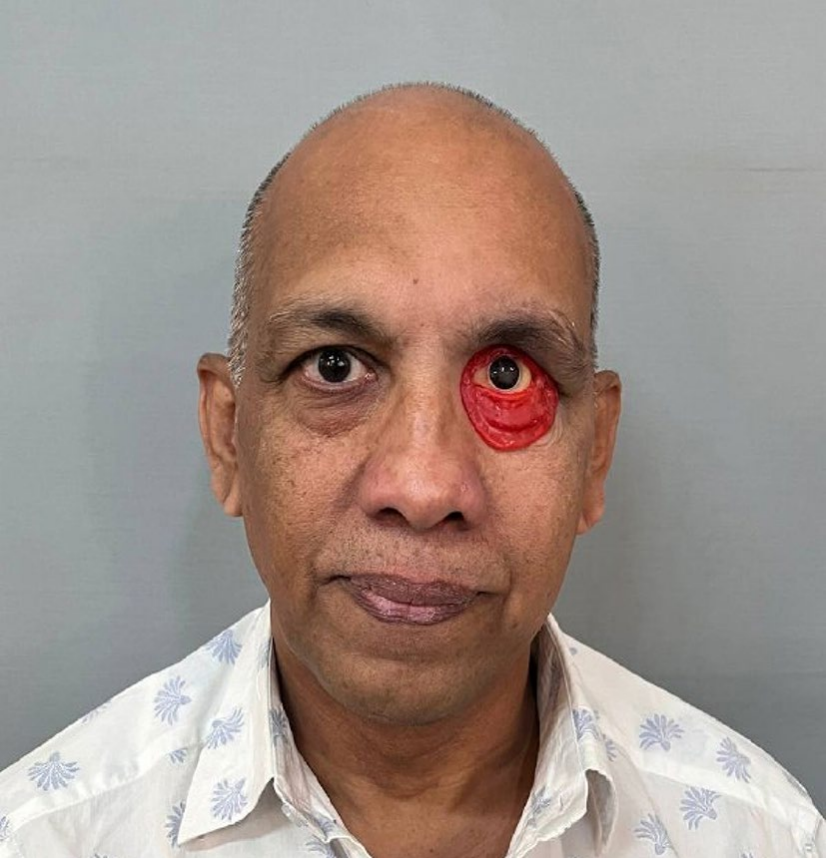
Wax try-in.

After the try-in (Fig. 8) was approved by the patient, flasking, dewaxing, and packing of the maxillofacial silicone was done. Heat temperature vulcanised silicone (technovent) was used for packing of the prosthesis. After 24 h, deflasking was completed. The final orbital prosthesis was finished. Extrinsic shading was done to give more natural appearance to the prosthesis and the extrinsic sealant applied for setting.

**Fig. 9:**
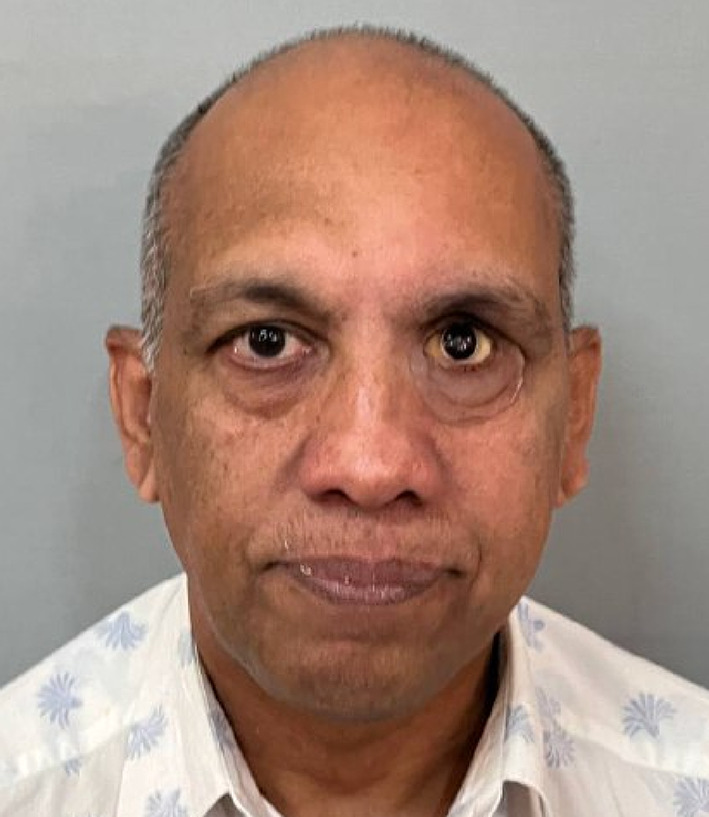
Post-op orbital prosthesis insertion.

**Fig. 10:**
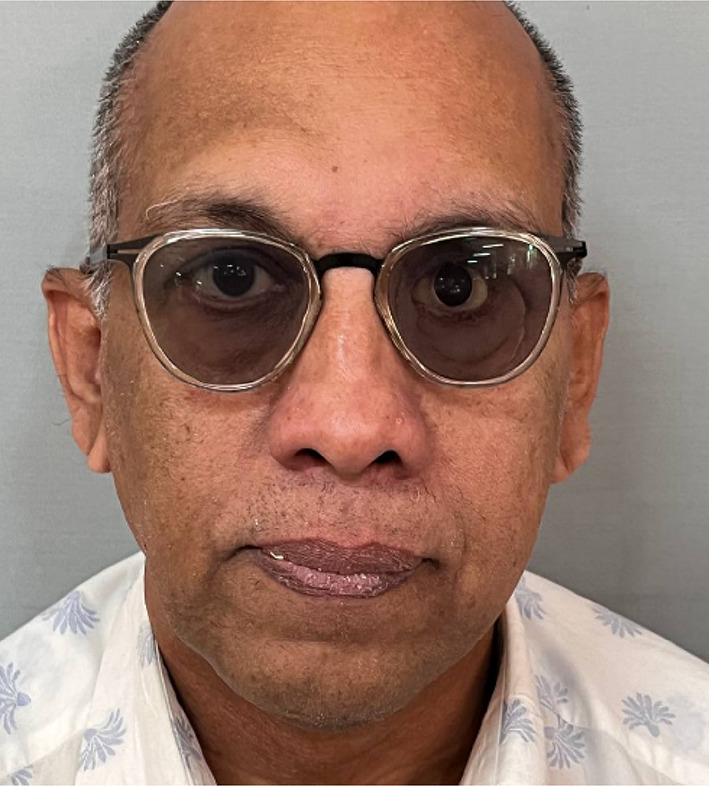
Spectacles used for added retention and masking the edges of the prosthesis.

The finished prosthesis was delivered to the patient (Fig. 9) and post-insertion instructions given. Although the prosthesis was retentive taking advantage from the tissue undercuts, patient was also advised to wear spectacles for masking the edges of the prosthesis **(**Fig. 10). Before starting the treatment, patient was provided with written informed authorization. Follow-up observations were made after one week and two months. Patient expressed pleasure with the prosthesis’ retention and the appearance.

## Results

**Fig. 11:**
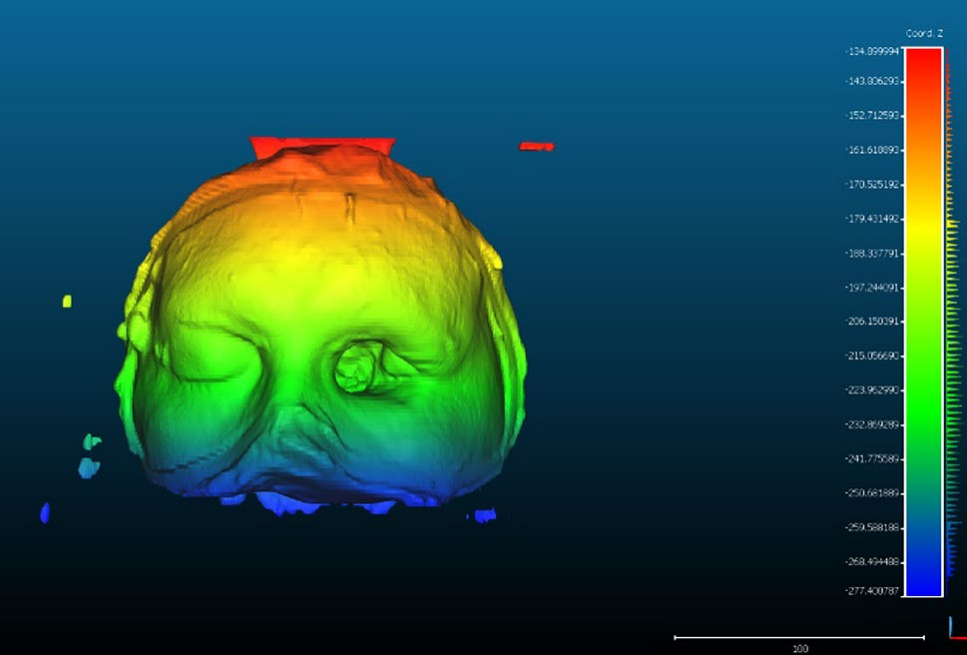
Computed Tomography (CT) face scan aligned with the photogrammetry scan data sing cloud compare(CloudCompare v2.6.1 https://www.danielgm.net/cc/).

The photogrammetry scan data (Fig. 11) was then aligned with the computed tomography (CT) scan of the face and evaluated for accuracy, completeness, and resolution, using Cloudcompare software. The results of this data overlay are shown in the Table 2; Fig. 11. Table 2; Fig. 11 provide a comparative analysis of photogrammetry scan data (using a DSLR camera at 90p) and computed tomography (CT) scan data, evaluated using Cloudcompare software. The comparison focuses on three key parameters: accuracy, completeness, and resolution, along with the maximum deviation defect.

In terms of accuracy, the CT scan data demonstrates higher precision with a value of 1.02, while the photogrammetry scan shows slightly lower accuracy at 1.32. This suggests that the CT scan more accurately captures facial details. For **completeness**, the photogrammetry scan performs marginally better, achieving a 97.67% completeness compared to 97.43% for the CT scan, indicating that the photogrammetry captured a slightly larger portion of the facial surface. However, in terms of resolution, the CT scan far outperforms photogrammetry, with a resolution value of 32.58 compared to 13 for the DSLR scan. This means that while the CT scan captures finer details, the photogrammetry scan has a lower level of detail.

These results highlight the trade-offs between the two methods: while photogrammetry offers good completeness and acceptable accuracy, CT scans deliver higher precision and much finer detail, making them preferable for applications where resolution is critical. Specifically, we compared the 3D-printed resin model against the original CT scan data to assess its dimensional accuracy, surface detail reproduction, and any deviations or distortions. This additional analysis provides a more comprehensive understanding of the overall workflow, from scanning to physical reproduction, ensuring that both the digital and physical models meet the required accuracy standards.

**Table 2:** Comparative analysis of CT scan data and photogrammetry data using Cloudcompare software.

Comparative results of CT scan data and photogrammetry	Accuracy	Completeness	Resolution
	Maximum deviation defect		
DSLR 90p	1.32	97.67	13
CT scan data	1.02	97.43	32.58

## Discussion

For many years, stock or custom ocular prostheses have been used to replace missing eyes. But frequently, especially for people who have lost ocular structures due to orbital evisceration or orbital enucleation, a custom-made orbital prosthesis that offers a more precise and pleasing aesthetic appearance is indicated^7^.

Conventional orbital prosthesis manufacturing techniques entail multiple complex steps that are costly, time-consuming, and stressful for the patient, and rely on the maxillofacial team’s, the prosthodontist, and maxillofacial technician’s expertise. Although CAD CAM technology has advanced in many fields of dentistry, there has been little application of digital approaches in cranial and maxillofacial rehabilitation^8^. Prosthetics made using modern manufacturing techniques and 3D scanning have many advantages over manual methods. Technology can lower costs and labor while improving patient comfort, customization, and satisfaction during the treatment. However, a large portion of the existing research outlining the benefits of this technology is predicated on the utilization of comparatively costly 3D printers and scanners. This can make it more difficult to apply them in therapeutic settings. The emergence of advanced computer software and the widespread availability of smartphones with high-quality cameras have created the opportunity to perform high-quality scanning at a relatively low cost when compared to high-end scanners, such as those used in photogrammetry techniques. Using 3D printing instead of conventional fabrication methods can significantly reduce the time needed to create a replica wax mold. Traditional methods, such as manual sculpting and casting, often take **days to weeks** depending on the complexity of the mold. Sculpting intricate details by hand is a labor-intensive process, requiring skilled artisans to carefully refine the surface, which can be quite time-consuming. Additionally, creating a mold using conventional casting methods, such as silicone or plaster molding, involves multiple steps, including mold preparation, curing, and wax casting, which further extends the timeline. In comparison, **3D scanning methods** offer an even faster alternative. The facial scanning process using photogrammetry or laser scanning typically takes **5–30 min** to complete, depending on the resolution and equipment used. The data is then processed digitally, and the 3D model is generated in **a few hours**, with no need for waiting on materials to set or harden. The total time for creating a digital mold through 3D scanning and 3D printing can often be completed within **1–3 days**, significantly reducing the total fabrication time compared to conventional methods.

Thus, while newer conventional methods have greatly reduced time consumption, **3D scanning still provides a faster overall workflow**, particularly for capturing and processing detailed facial data.

In contrast, 3D printing offers a much faster workflow. A digital model can be prepared and processed within **hours to a couple of days**, and the actual printing time typically ranges from **a few hours to a day**, depending on the printer and model size. This bypasses many of the manual labor steps required in conventional fabrication. Once the printed model is complete, minimal post-processing is needed compared to traditional methods, saving additional time.

Overall, using 3D printing for part of the model (such as the back surface) can cut the total fabrication time to **1–3 days**. Compared to the **1–2 weeks** often required for conventional methods, this can represent a time savings of **50–70%**. The reduction in manual labor, the precision of 3D printing, and the quicker turnaround make it a highly efficient alternative, especially for projects involving complex molds.

In rural areas, where access to advanced technology can be limited, there are challenges with using digital fabrication methods for making maxillofacial prostheses. Issues like the availability, accessibility, and cost of software can pose significant obstacles. To address these challenges, we’ve developed a hybrid approach for manufacturing these prostheses. (Table 3**)** This method combines the strengths and weaknesses of both digital and traditional fabrication techniques.

**Table 3:** Illustrates the algorithm for conventional, digital and hybrid workflow.

	Workflow	Clinical efficacy	Time	Cost effectiveness	Edge quality and marginal adaptation	Aesthetic outcomes	Material characteristics
CONVENTIONAL FABRICATION	Traditional Impression making and multiple try-ins required	labor intensive and complex steps	Time intense	Cheaper, when compared to digital technique	Good	Patient relies on the skills of Prosthodontist	Medical grade silicone
*HYBRID*	***3D capture of facial topography*** ***(photogrammetry)***	***Excellent; contactless*** ***Semi-automated***	***Less time consuming compared to conventional fabrication***	***Cuts off the additional digital fabrication costs***	***Acceptable***	***Acceptable***	***Medical grade silicone***
DIGITAL FABRICATION	3D capture of facial topography	Excellent; sometimes challenging and prone to errors	Lesser time required	Costlier compared to traditional	Reasonably low	Acceptable	No material is clinically approved for direct fabrication

In maxillofacial prosthodontics, advanced digital technology has showed potential in replacing several phases in the conventional workflow of designing and constructing facial prosthesis. Traditional impression, modelling, and production procedures are likely to be replaced by digital equivalents since the development of 3D scanners, software, and RP technology^9–11^. In most cases, the hybrid technique with a digital 3D printed silicone injection mould, using standard procedures and followed by manual color individualization, is necessary to achieve aesthetic results for the final orbital prosthesis that are comparable to those produced with the analogical path^5,12^.

## Conclusion

In conclusion, the present study offered a unique semi-automated process that can construct customised orbital prostheses faster and with less technical expertise. The ability to preserve the 3D modelling data has another benefit because it enables the reuse of the data in the event that the patient’s ocular prosthesis is misplaced or harmed. By making it simpler for patients to obtain high-quality, custom-made orbital prostheses, the proposed method contributes to their enhanced professional and societal acceptance.

## Data Availability

The data will be available on Figshare with the following DOI 10.6084/m9.figshare.25965469 (by the corresponding author)
